# On the Potential Function of Type II Arabinogalactan *O-*Glycosylation in Regulating the Fate of Plant Secretory Proteins

**DOI:** 10.3389/fpls.2020.563735

**Published:** 2020-09-10

**Authors:** Georg J. Seifert

**Affiliations:** Department of Applied Genetics and Cell Biology, Institute of Plant Biotechnology and Cell biology, University of Natural Resources and Life Sciences Vienna, Vienna, Austria

**Keywords:** FLA4, secretion, traffic, golgi apparatus, protein quality control

## Abstract

In a plant-specific mode of protein glycosylation, various sugars and glycans are attached to hydroxyproline giving rise to a variety of diverse *O-*glycoproteins. The sub-family of arabinogalactan proteins is implicated in a multitude of biological functions, however, the mechanistic role of *O-*glycosylation on AGPs by type II arabinogalactans is largely elusive. Some models suggest roles of the *O-*glycans such as in ligand-receptor interactions and as localized calcium ion store. Structurally different but possibly analogous types of protein *O-*glycosylation exist in animal and yeast models and roles for *O-*glycans were suggested in determining the fate of *O-*glycoproteins by affecting intracellular sorting or proteolytic activation and degradation. At present, only few examples exist that describe how the fate of artificial and endogenous arabinogalactan proteins is affected by *O-*glycosylation with type II arabinogalactans. In addition to other roles, these glycans might act as a molecular determinant for cellular localization and protein lifetime of many endogenous proteins.

## Structure of Type II Arabinogalactan *O-*Linked Glycans and Types of Arabinogalactan Proteins

**Figure 1:**
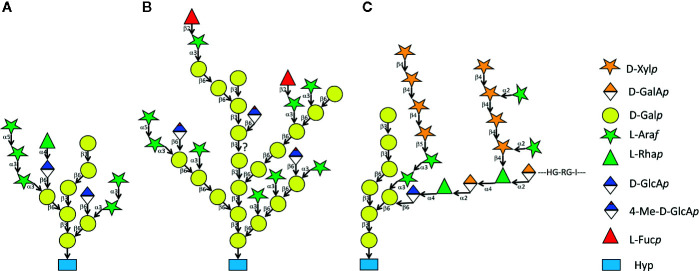
Three variants of type II AG. Structure **(A)** was determined from the synthetic AGP (Ala-Hyp)51-EGFP expressed in and secreted from *Nicotiana tabacum* Bright Yellow 2 suspension cells (Tan et al., 2004). Among other techniques, the Hyp-glycan was analyzed using nuclear magnetic resonance spectrometry. Structure **(B)** is one of many possible models derived from enzymatic fragmentation and electrophoretic as well as mass spectrometric analysis of AGP bulk-purified from *A. thaliana* leaves (Tryfona et al., 2012). Stucture **(C)** shows a type II AG sub-structure linked to APAP1/AGP57C and to arabinoxylan and pectic rhamnogalacturonan I (Tan et al., 2013).

Eukaryotic cells secrete a multitude of both *N-*linked and *O-*linked glycoproteins. In plants, the group of hydroxyproline-rich glycoproteins (HRGPs) that are *O-*glycosylated at hydroxyproline (Hyp) residues comprise extensins (EXTs), arabinogalactan proteins (AGPs), and proline-rich proteins (PRPs), however, genome wide analyses reveal a continuum of *O-*glycosylation sequence motifs (see below) in various combinations and together with distinct protein domains (Showalter et al., 2010; Johnson et al., 2017). EXTs are *O-*glycosylated by short linear arabinans while AGPs are *O-*glycosylated with branched type II arabinogalactans (AGs) (**Figure 1**). The little characterized PRPs are generally believed to carry only minimal glycans (Showalter et al., 2010), however there can be large heterogenity in the overall extent of *O*-glycosylation of individual PRPs (Hijazi et al., 2012). Individual type II AG structures vary between species and between tissues in the same species however, their common feature is a backbone of β-1-3 linked galactan that contains β-1-6 galactan branches and further modification by α-1-3 linked arabinofuranose (**Figure 1**). Many type II AG structures contain glucuronate or 4-methyl-glucuronate linked to galactose in β-1-6 as well as terminal modifications by L-rhamnose and L-fucose. An important aspect of the β-1-3-galactan backbone is its specific binding to β-glucosyl Yariv reagent, a diazo-dye that has profusely been used for the isolation and identification of AGPs and for *in vivo* interference with AGP function (Kitazawa et al., 2013; Paulsen et al., 2014). Another diagnostic tool of type II AG are carbohydrate reactive monoclonal antibodies. The epitopes on type II AG recognized by some of these highly specific and sensitive probes have been elucidated by synthetic oligosaccharides and glycan arrays (Pattathil et al., 2010; Ruprecht et al., 2017). While type II AG was detected by monoclonal antibodies and β-glucosyl Yariv reagent in many plant species, occasionally there have been descriptions of other types of *O*-glycosylation of AGPs such as the heterogeneous glycans found on AGP31 recognized by peanut lectin (Hijazi et al., 2012) and the type III AG found on some allergens (Leonard et al., 2005; Leonard et al., 2010).

Early studies of the protein sequence of bulk-purified HRGPs globally revealed that EXTs contained stretches of contiguous Hyp residues preceded by Ser/Thr (S/T[O]_3-5_) while in AGPs, Hyp residues were typically clustered but separated by Ser/Thr/Ala residues [A/S/TP]_n≥2_ (Showalter, 1993; Chen et al., 1994; Du et al., 1994). The repetitive sequence motifs are thought of as the functional units that inform the post-translational attachment of specific O-glycans and are termed glycomodules or more specifically, EXT modules in the former case and AGP modules in the latter. The function of glycomodules as attachment sites of *O*-glycans was confirmed by expressing artificial AGPs and EXTs in plants (Shpak et al., 1999; Estevez et al., 2006), and led to the Hyp contiguity hypothesis which suggests that contiguous Hyp residues are modified by short linear arabinans and isolated Hyp residues are galactosylated and subsequently modified by type II AG (Kieliszewski, 2001). Additional studies have further refined the empirical rules for protein *O-*glycosylation in plants (Shimizu et al., 2005; Canut et al., 2016). While some prolyl 4-hydroxylase (P4H) isoforms have previously been implicated in the generation of contiguous Hyp residues in EXTs and with root hair formation (Velasquez et al., 2015), and virus induced gene silencing of P4H isoforms in *Solanum lycopersicum* suggested a growth controlling role of unidentified HRGPs (Fragkostefanakis et al., 2014), it is presently unclear if individual PH4 isoforms specifically act on EXT or AGP modules. At any rate, prolyl hydroxylation might act as one early acting mechanism in the determination of the position and extent of O-glycosylation.

*Classical AGPs* and short *AG-peptides* consist of a protein backbone that essentially contains a stretch of discontiguous Hyp residues mostly *O-*glycosylated by type II AG considerably varying in size and fine structure while *hybrid AGPs* such as fasciclin like AGPs (FLAs) and phytocyanin AGPs (PAGs, some of which are called early nodulin likes—ENODLs) contain additional protein domains. However, based on the occurrence of AGP modules, there might be an even larger variety of structurally diverse proteins that carry type II AG. A large proportion of the glycosylphosphoinositol-lipid anchored proteins (GPI-APs) of *Arabidopsis thaliana* either are AGPs, FLAs or PAGs or carry AGP modules in their protein sequence (Borner et al., 2002; Elortza et al., 2006). But also, many other secreted or plasma membrane proteins might by *O-*glycosylated. For instance, a simple sequence pattern suggested to lead to efficient arabinogalactosylation (Shimizu et al., 2005) occurs in nearly 1,200 secretory proteins^1^ of *A. thaliana*. Trans-membrane proteins such as receptor like kinases (RLKs) can carry both EXT modules and AGP modules. Accordingly named RLK families are proline-rich extensin*-*like receptor kinases (PERKs) and EXT-like RLKs (Shiu and Bleecker, 2003). Potential *O-*glycosylation motifs in the extracellular domain close to the trans-membrane region also exist in most Strubbelig family RLKs (Eyuboglu et al., 2007), in five members of the multifunctional Somatic Embryogenesis Related Kinase (SERKs) family (Li, 2010) and in individual representatives of many other RLK families and in other secreted protein families. However, there is no simple way to predict or experimentally determine *O-*glycosylation. Clearly, at present the *O-*glycoproteome is strikingly under-annotated.

## Molecular Biology of Type II Arabinogalactan Biosynthesis

Consistently, the CAZy database of carbohydrate active enzymes (Henrissat et al., 2001) contains hundreds of orphaned both glycosyltransferases (GTs) and -hydrolases (GHs) many of which can be expected to act in protein *O-*glycan biosynthesis and remodelling, respectively. Still, the recent elucidation of the biosynthetic machinery of type II AG is a success story. Type II AG-biosynthesis requires enzymes to generate the necessary nucleotide sugars and GTs to specifically incorporate the sugars into the glycan. Most if not all *A. thaliana* genes encoding nucleotide sugar metabolic enzymes required for AGP biosynthesis are known (Seifert, 2004; Reiter, 2008; Bar-Peled and O’Neill, 2011) and as of now, at least eighteen different GT genes have been associated with type II AG biosynthesis (Knoch et al., 2014; Nguema-Ona et al., 2014; Showalter and Basu, 2016a; Li et al., 2017; Suzuki et al., 2017). For instance, eight family 31 glycosyltransferases (GT31) called GALT2 to -6 as well as HPGT1 to -3, catalyse the galactosylation of isolated Hyp residues on short peptides *in vitro* (Basu et al., 2013; Basu et al., 2015a; Basu et al., 2015b; Ogawa-Ohnishi and Matsubayashi, 2015; Showalter and Basu, 2016b). The backbone is further extended by β-1-3 galactosyltransferase, with the GT31 KNS4/UPEX1 which is required for pollen exine development (Suzuki et al., 2017) and another closely related *GT31* locus showing activity *in vitro* (Qu et al., 2008). The GT31 and GT29 loci called *GalT31A* and *GalT29A* might cooperate to introduce and elongate the β-1-6 galactan branches (Dilokpimol et al., 2014). Various AGP specific galactosyl transferases are essential for processes such as embryogenesis, root elongation, salt tolerance, and seed coat mucilage formation. In addition, the ectopic expression of type II AG biosynthetic GalTs can have dramatic and complex developmental effects (Geshi et al., 2013; Qin et al., 2013; Basu et al., 2015a; Basu et al., 2015b; Ogawa-Ohnishi and Matsubayashi, 2015; Basu et al., 2016; Qin et al., 2016). The molecular mechanism of the modification of side chains by arabinofuranosyl residues is still elusive. Interestingly, the GT77 locus *RAY1* which is related to the EXT specific β-1-3-arabinofuranosyl transferase XEG113 was proposed to generate a β-1-3-arabinosyl linkages on a Yariv reagent-precipitable polymer. The *ray1* mutant displayed a reduction of arabinose in type II AG and showed abnormal growth. However, as was previously noted, arabinose in type II AG is α-linked and the precise biochemical role of RAY1 in AGP biosynthesis remains to be clarified (Showalter and Basu, 2016a). By contrast, several type II AG specific β-1-6-glucuronosyl transferases are included in family GT14—called GlcAT14-A, -B, and -C. The *glcat14* single and multiple mutants in these three loci showed a variety of developmental defects as well a reduction of AGP-bound calcium (Knoch et al., 2013; Dilokpimol and Geshi, 2014; Zhang et al., 2020). Finally, two members of family GT34 called FUT4 and FUT6 redundantly acted in the α-1-2 fucosylation of type II AG (Liang et al., 2013; Tryfona et al., 2014). The *fut4 fut6* double mutant showed a strong reduction in type II AG linked fucose, and oversensitivity to 150 mM NaCl compared to wild type controls. While the above mentioned studies show that type II AG structure depends on a series of biosynthetic reactions, recent data show that these *O-*glycans are also subject to remodeling *in muro* by glycoside hydrolases (GHs) such as exo-β-1-3-galactosidases (Nibbering et al., 2020), β-L-arabinopyranosidases (Imaizumi et al., 2017) and β-glucuronidase (Eudes et al., 2008). As described for mutants in loci coding for type II AG specific GTs, mutations in type II AG active GH loci caused aberrant growth phenotypes. The mechanism how type II AG structure affects development is unclear.

There exist several critical differences between *N-*linked and *O-*linked glycosylation. While *N-*glycosylation begins in the endoplasmatic reticulum (ER) as co*-*translational *en bloc* transfer of a preformed oligosaccharide that is subsequently modified in the Golgi, type II AG biosynthesis is entirely Golgi-localized and depends on the hydroxylation of specific prolyl residues in the cis-Golgi followed by galactosylation of isolated Hyp residues and sequential build-up of type II AG in subsequent Golgi compartments. In a recent proteomics study, several P4H isoforms were found the cis and medial Golgi compartment, while HPGT2 was localized in the medial Golgi (Parsons et al., 2019). Some type II AG biosynthetic enzymes such as AtGALT31A have also been colocalized with exocyst protein Exo70E2 in a novel compartment, a finding whose significance remains to be investigated (Poulsen et al., 2014). Additional complications in the secretion of AGPs are due to the fact that most of them are GPI-anchored and use a specific trafficking machinery (Baral et al., 2015; Muniz and Riezman, 2016; Lopez et al., 2019).

## What is the Function of Type II Arabinogalactan *O-*Linked Glycans in Plants?

But what is the function of type II AG *O-*glycosylation? AGPs have been implicated in diverse biological functions (Showalter, 1993; Nothnagel, 1997; Sommer-Knudsen et al., 1998; Showalter, 2001; Seifert and Roberts, 2007; Ellis et al., 2010; Nguema-Ona et al., 2012; Lamport et al., 2014; Huang et al., 2016). The conspicuous labelling patterns and *in vitro* effects of type II AG-glycan specific monoclonal antibodies as well as the various dramatic effects triggered by Yariv reagents (Yariv et al., 1962; Kitazawa et al., 2013; Paulsen et al., 2014) have led to the notion that the function of an AGP is entirely based on a direct function of its glycan with the protein moiety only acting as a template for glycan attachment. In the case of classical AGPs and AG-peptides that merely consist of highly glycosylated but otherwise intrinsically disordered protein backbones attached to GPI-anchors this obviously must be true. An intriguing possibility of how AGPs might act *via* their *O-*glycans was revealed in an amazing analytical tour de force of Tan and coworkers (Tan et al., 2013). The **APAP1/AGP57C** protein was found as a minor AGP fraction secreted by suspension cells and the *apap1* knock out mutants in *A. thaliana* showed a minor growth defect but substantial alterations in some cell wall polysaccharides’ compliance to sequential extraction. While the APAP1 backbone was unstructured apart from some AGP modules, and the core glycan structure resembled a typical AGP glycan it contained numerous novel crosslinks to both pectic rhamnogalacturonan I and hemicellulosic arabinoxylan (**Figure 1C**). This structure revealed that type II AG can be crosslinked to other cell wall polysaccharides and opened many intriguing questions such as the possibility that such crosslinks might be part of others AGPs.

Another example which showed that the glycan of an AGP was directly involved in its function was **xylogen**. This glycoprotein was biochemically isolated as a factor that induced tracheary element formation in *Zinnia elegans* cell cultures. Apart from three AGP modules and a GPI-anchor modification motif, xylogen contained a nonspecific lipid-transfer protein domain. In its active form, xylogen contained Hyp in its protein backbone and reacted with AGP-specific monoclonal antibodies as well as Yariv reagent. Chemical de-glycosylation of xylogen abrogated its *in vitro* inducive effect on transdifferentiation suggesting that the type II AG of xylogen was directly involved in its function (Motose et al., 2004), possibly by promoting or forming a ligand receptor interaction.

A remarkable effort to mechanistically define glycan function of classical AGPs has focused on the fact that many type II AGs contain glucuronic acid (**Figure 1**). According to this so called **Ca^2+^-capacitor hypothesis**, GPI-anchored classical AGPs can bind Ca^2+^ ions at a molar ratio of 1:1 per type II AG-subunit (**Figure 1A**) with a K_d_ of 5 µM and in a pH-sensitive fashion. Acidification of the apoplast was suggested to lead to a release of Ca^2+^ close to the plasma membrane to be available for import by Ca^2+^- channels and subsequent intracellular signaling. This hypothesis was employed to explain AGP function with respect to pollen tube growth and phyllotaxis and it was suggested as the mechanistic basis of the long standing acid growth theory (Lamport et al., 2014; Lamport et al., 2018; Lamport et al., 2020). On the one hand, the very low resting level of Ca^2+^ in the cytosol (ca. 0.2 µM) compared to much higher apoplastic levels and the Ca^2+^-storage capacity of vacuoles seemingly speaks against the requirement of a biosynthetically expensive extracellular Ca^2+^-reservoir. Furthermore, the model seems to under-emphasize the important controlling role of plasma membrane Ca^2+^ channels. On the other hand, a plasma membrane linked capacitor might not necessarily buffer against *global* apoplastic Ca^2+^-deficiency but rather provide an *unstirred layer* of Ca^2+^ ions, readily available for intracellular signaling at a relatively constant level (Gilliham et al., 2011). Moreover, the Ca^2+^-capacitor hypothesis does not exclude any other roles of type II AG. At any rate, the Ca^2+^-capacitor hypothesis is so far supported at the genetic level by mutant plants lacking several type II AG specific glucuronyl transferases (Knoch et al., 2013; Dilokpimol and Geshi, 2014; Lopez-Hernandez et al., 2020; Zhang et al., 2020).

A very productive area of AGP-related research is **floral development and sexual reproduction** (Pereira et al., 2016a; Su et al., 2020). Some classical AGPs and AG-peptides are required for normal pollen tube growth (Levitin et al., 2008; Pereira et al., 2016b) and in the light of the Ca^2+^-capacitor hypothesis it would be interesting to test if the pollen tube phenotype previously observed in triple mutants lacking some of the type II AG specific glucuronyl transferases (Zhang et al., 2020) is modulated by external Ca^2+^ levels. However, sometimes the glycan does not seem to be directly involved in the function of an AGP. Here one can quote another recent example for AGPs acting in sexual reproduction. It was shown that several PAGs from *A. thaliana* called early nodulin*-*like peptides (ENODLs11 to -15) acted in female guidance of the pollen tube and in sperm release. The receptor kinase FERONIA (FER) was previously shown to act in the same process and, intriguingly, ENODL14 physically interacted with FER. Significantly, this interaction took place in heterologous assays in yeast and *Escherichia coli* and thus did not require glycosylation of the AGP modules (Hou et al., 2016). In line with this finding, a protein from cotton named GhPLA1 that was highly homologous to ENODL14 stimulated somatic embryogenesis independent of its glycan also arguing against a direct role of the type II AG for the signaling function of this group of AGPs (Poon et al., 2012). This contrasted with another study on female to male signalling in flowers that showed that the type II AG fragment 4-Me-GlcA-β(1,6)-Gal termed AMOR was biologically active in determining pollen tube competence. It is not clear, however, whether AMOR, a common structure in type II AG (**Figure 1B**), is derived from an ENODL or any other of the many AGPs expressed in flowers (Mizukami et al., 2016). In a fine glycobiological structure to function study it was shown that methylation, anomeric beta form and (1-6) linkage as well as a pyranose (irrespective the sugar) at the reducing end of the disaccharide were essential for AMOR function (Jiao et al., 2017). However, a larger number of Gal residues at the reducing end did not reduce activity (Mizukami et al., 2016), hence the AMOR disaccharide might in principle also act as the terminal part of a larger glycan structure either released from or as integral part of an AGP.

**Figure 2:**
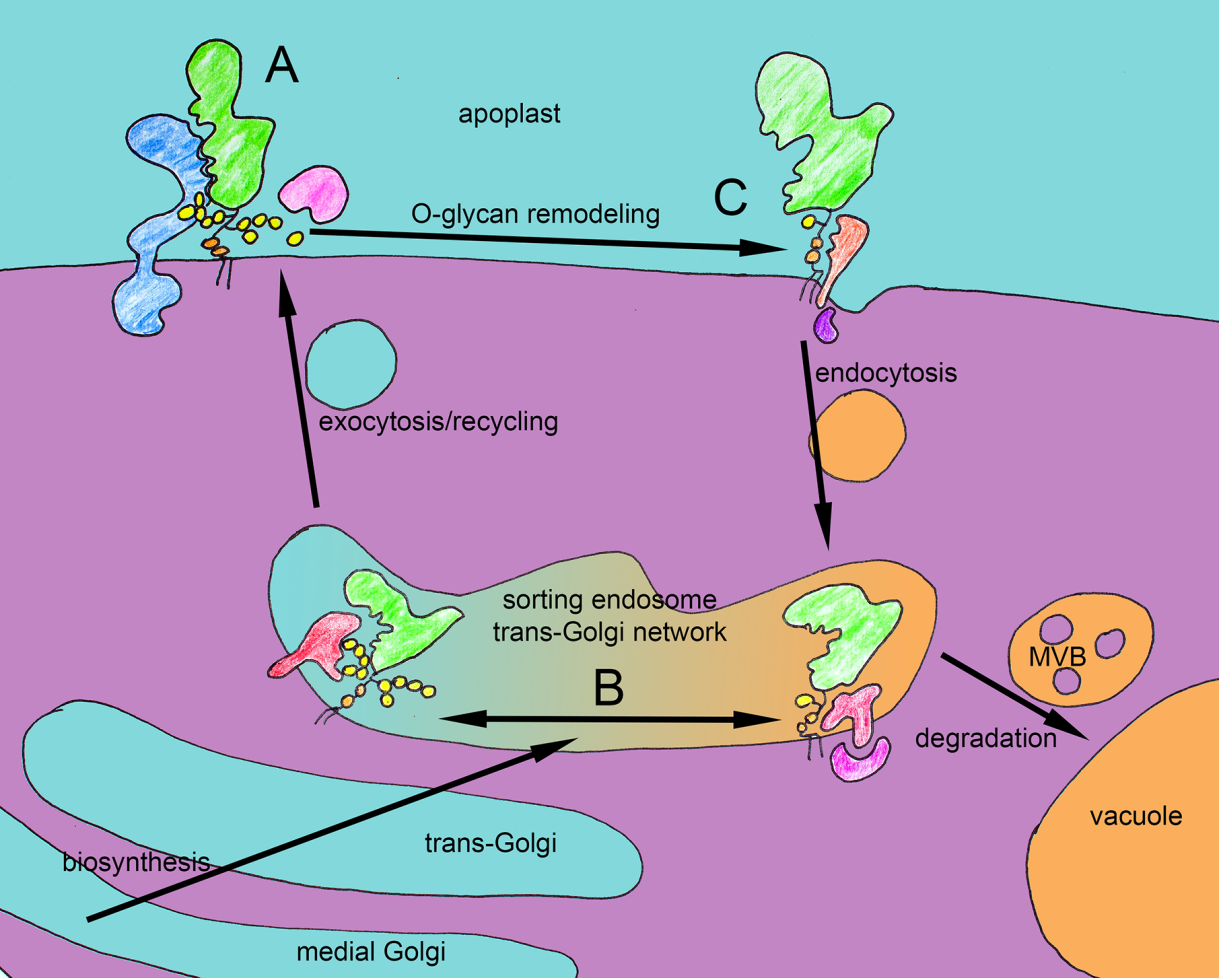
Hypothetical functions of *O-*glycans of FLA4 in trafficking and receptor binding. Three potential scenarios that might explain recent genetic and cell biological observations. **(A)** The *O-*glycan (yellow) might be required for interaction between FLA4 (green) and FEI receptor kinases (blue). **(B)** In the secretory pathway the *O-*glycan might be checked by a system of different cargo receptors (red) that either bind fully or incompletely *O-*glycosylated FLA4 and their cytosolic adaptors (purple). While the fully glycosylated FLA4 molecules are rapidly transported to the cell surface *via* exocytosis, the hypoglycosylated species are targeted for destruction to the vacuoles *via* multivesicular bodies. **(C)** Full *O-*glycosylation might stabilize FLA4 at the cell surface and as binding partner for receptor kinases while its apoplastic remodeling by glycanases (pink) might make the protein competent to be recruited by endocytotic cargo receptors. The number and identity of the involved molecular components are unknown and despite their colorful depiction, the symbols for receptors and cytosolic adaptors represent “black boxes” at the moment.

Studying single and multiple mutants in AGP protein backbone encoding genes can be genetically specific but this approach does not allow to derive any glycan-specific roles because it cannot be excluded that other AGP protein backbone encoding genes that are still expressed in the mutants carry the same or different *O-*glycans. Hence complementary to that approach, mutant models defective in AGP-glycosylation have recently been established. Mutants in the *A. thaliana* *UDP-GLUCOSE 4-EPIMERASE 4 (UGE4)* locus (Seifert et al., 2002) defective in the *de novo* biosynthesis of UDP-D-galactose, showed a quantitative reduction and qualitative alteration of AGPs (Ding and Zhu, 1997; Andeme-Onzighi et al., 2002; Nguema-Ona et al., 2006). The *uge2 uge4* double mutant showed a severe general growth phenotype for which defective glycosylation of AGPs might be responsible (Rösti et al., 2007). While the phenotypic similarity between Yariv-treated roots (Willats and Knox, 1996; Ding and Zhu, 1997) and *uge4* mutants was intriguing, mutants in nucleotide sugar metabolism can affect many different biopolymers and type II AG specific GT mutants promised higher specificity. The GALT2 and -5 and the HPGT1 to -3 showed *in vitro* activity to galactoslate AGP module-like Hyp containing peptides. Single mutants, in all five loci showed a reduction in the content of Yariv reagent-precipitated AGPs by 11 to 42%, while *galt2 galt5* double mutants, showed a 58% reduction (Basu et al., 2013; Basu et al., 2015a; Basu et al., 2015b) and the *hpgt1 hpgt2 hpgt3* triple mutant showed a reduction by more than 70% (Ogawa-Ohnishi and Matsubayashi, 2015). In principle, the reduced content of AGPs in the above cited studies can be explained by the way AGPs are binding to Yariv reagent *in vitro via* the β-1-3-galactan chains in their *O-*glycans (Kitazawa et al., 2013; Paulsen et al., 2014). However, another explanation for the observed reduction could be a **role of galactosylation for the stability and lifetime** of AGPs *in vivo*. While not a phenocopy of Yariv treatment, both the *galt2 galt5* double mutant and the *hpgt1 hpgt2 hpgt3* triple mutant displayed a root and seed coat mucilage phenotype apparently identical to *salt overly sensitive5* (*sos5-1*), a mutant in the *FASCICLIN LIKE ARABINOGALACTAN PROTEIN 4 (FLA4)* locus (Johnson et al., 2003). The *sos5-1* mutant had been previously identified in a mutant screen for salt oversensitive root growth (Shi et al., 2003) and was subsequently found to act in a linear genetic pathway with two redundantly acting leucine-rich receptor kinases (LRR-RLK) named FEI1 and FEI2 (Xu S. L. et al., 2008). Apart from displaying slightly stunted root growth and massive root growth defects at elevated levels of sugar or salt, both *sos5* and *fei1 fei2* showed a seed coat mucilage adhesion defect that interacted non*-*additively with mutants causing aberrant mucilage pectin structure (Griffiths et al., 2014; Griffiths et al., 2016). The genetic interaction between *sos5* and *fei1 fei2* might in principle suggest that FLA4 and the FEI proteins directly interact in a membrane localized complex. However, apart from *in silico* docking (Turupcu et al., 2018), experimental evidence for colocalization of FLA4 and the FEIs *in vivo* or their physical interaction *in vitro* is presently lacking. When the genetic interaction between the Hyp-specific galactosyltransferase mutants *galt2 galt5* and *hpgt1 hpgt2 hpgt3* on the one hand and *sos5* and *fei1 fei2* on the other hand was tested it was found that the transferase mutants acted non*-*additively with both *sos5 and fei1 fei2* suggesting a linear genetic pathway (Basu et al., 2016). This fascinating finding suggested that glycosylation of AGPs is required for the genetic function of *SOS5* and *FEI1 FEI2* (Basu et al., 2015b; Ogawa-Ohnishi and Matsubayashi, 2015; Basu et al., 2016; Showalter and Basu, 2016b). In principle this notion complies with the model proposed by Showalter and coworkers (Basu et al., 2016) in which FLA4 and the FEI RLKs form a signaling complex with the type II AGs of FLA4 essential for FEI signaling (**Figure 2A**).

However, while this model clearly fits the genetic data, the presently available molecular and cell biological data are also explained by an alternative model, as explained below. In order to study the localization and intracellular trafficking of a functional hybrid AGP, we have generated a fluorescent protein*-*tagged FLA4-citrine (F4C) which fully complemented the *sos5* mutant alleles. In contrast to classical AGPs, FLA4 lacks typical (XP)_n≥2_ repeats in its sequence. However, we showed that F4C carries the type II AG specific *O-*glycan epitopes reacting with the JIM13 and LM14 monoclonal antibodies (Xue et al., 2017). The precise position and structure of FLA4’s *O-*glycan remain to be determined, however, to investigate its functional role all the clustered proline residues that were candidates for *O-*glycosylation were replaced by alanine residues. This manipulation resulted in strongly reduced reactivity of the protein with with LM14 and abolished JIM13 labelling which indicated that most of the type II AG was absent from the mutated protein. This led to dramatically altered protein localization of F4C. The “wild type” F4C that contains six and four clustered proline residues in its central and its C-terminal proline-rich domain, respectively, was predominantly localized at the plasma membrane from where it was partially released to the apoplast, and it also underwent endocytosis and recycled to the plasma membrane. By contrast, the F4C_P1234A probe in which these clustered proline residues were replaced by alanine residues was mostly located in intracellular compartments reactive with molecular markers of the endosome/trans Golgi network and the pre-vacuolar compartment. Moreover its overall abundance was strongly reduced (Xue et al., 2017). Importantly however, the *F4C_P1234A* construct fully complemented the *sos5* mutant suggesting that *FLA4* did not require its proline-rich domains for genetic function. In combination, the genetic and cell biological observations are compatible with an important role of the FLA4 *O-*glycans for correct intracellular trafficking and protein lifetime. Hypothetically, the degree of type II AG modification of FLA4 might decide between passage to the plasma membrane of a highly glycosylated protein or degradation in the lytic vacuole of a hypoglycosylated form (**Figure 2B**). As FLA4 is normally expressed at a low level then the *O-*glycan-dependent degradation might completely block its passage to the plasma membrane and prevent the speculative interaction between the globular Fas1 domains of FLA4 with the FEI ectopic domain (Turupcu et al., 2018). Overexpression of *FLA4* might lead to a bypass of hypoglycosylated protein to the plasma membrane. This scenario would explain both the linear genetic interaction between the glycosylation mutants and *sos5* or *fei1 fei2*, and the complementation of *sos5* by hypoglycosylated FLA4.

While the type II AG is a relatively minor part of the FLA4 holoprotein, the AG peptide **AtAGP21** consists of nothing but a heavily *O-*glycosylated (i.e. a 25-30 kDa type II AG on each of the three Hyp residues) 12-aa peptide attached to a GPI anchor. AGP21, fused to a fluorescent protein*-*tag was efficiently targeted to the plasma membrane and the apoplast. Similar to FLA4, a proline to alanine mutant version of YFP-AGP21 was not only much less abundant but also was mostly retained in the cytoplasm (Borassi et al., 2020). However, it did not complement the *agp21* root hair phenotype. So, *O-*glycosylation of AGPs is required for both correct signalling and plasma membrane localization of endogenous proteins. Type II AG might also affect partitioning of GPI-anchored proteins between the plasma membrane and plasmodesmata, because YFP fused to the GPI-anchor signal from AGP4, was efficiently targeted to plasmodesmata, but the inclusion of an AGP module (SPAPS) immediately preceding the GPI-modification signal of AGP4 led to a partial distribution to plasma membrane outside plasmodesmata (Zavaliev et al., 2016). Apparently, type II AG might not only act as Ca^2+^-reservoir at the plasma membrane, as crosslinkers in the cell wall matrix or as source of apoplastic oligosaccharide signals, they might also regulate intracellular trafficking between the plasma membrane and vacuolar degradation. I speculate that there might be a **cellular checkpoint for *O-*glycosylation in a post-Golgi compartment**, potentially the endosomal/trans Golgi network compartment sometimes termed sorting endosome (**Figure 2B**). This machinery might recognize structures in misfolded or insufficiently *O-*glycosylated protein regions and shuttle them towards vacuolar degradation. However, alternatively, the presence of type II AG might provide a positive signal for secretion. Evidence that this might be the case came from the expression of recombinant artificial and heterologous AGPs.

**Artificial AGP** genes were initially generated to investigate glycomodules leading to different types of *O-*glycosylation. SynAGPs typically consisted of secreted GFP fused to variable (XP)_n≥2_ repeats. However, it was soon realized that AGP modules as short as the SPSP tetrapeptide, introduced into non*-*plant proteins such as GFP, interferon α2b or human growth hormone, dramatically increased secretion of the recombinant proteins compared to their unmodified precursors (Estevez et al., 2006; Xu et al., 2007; Xu J. et al., 2008; Xu and Kieliszewski, 2011). To cite a recent addition to these studies, the effect of long EXT modules and AGP modules on the secretion of GFP was tested in hairy root cultures (Zhang et al., 2019). Fusion of either type of glycomodule to GFP resulted in a more than 50-fold higher presence of *O-*glycosylated GFP fusion protein compared to non*-*glycosylated GFP in supernatants of 12-day old cultures. From kinetic observations, the authors concluded that EXT-type *O-*glycosylation might be a slower process than AGP-type *O-*glycosylation and in consequence more rate limiting for secretion. Interestingly, nitrogen depletion led to a dramatic increase of glycosylation of the AGP modules. When such observations made with fluorescent protein fusion constructs are extrapolated to endogenous AGPs they suggest that developmental or environmental control of type II AG biosynthesis might influence the cellular fate of many AGPs. Hence, type II AG might directly interact with other macromolecules acting in a structural-mechanical network together with other cell wall biopolymers, they might act in signaling, either as part of the glycoprotein they are attached to or as small glycan fragment. However, type II AG attached to hybrid AGPs such as FLA4 might also act indirectly by controlling intracellular trafficking and stability of the apoprotein. Because very little about the latter possibility is known in plant cell biology I will describe several scenarios from other kingdoms where *O-*glycosylation affects protein fate.

## *O-*Glycosylation Controls Protein Fate in Animals and Fungi

In all eukaryotes, *O-*glycans have been associated with many different biological functions. Here, I will focus on the involvement of *O-*glycans in the control of protein fate in animals and yeast. In mammals, many proteins are modified by GalNAc containing mucin*-*type *O-*glycans (Hansen et al., 1998) using a Golgi localized set of glycosyltransferases (Burchell et al., 2018; Steentoft et al., 2019). Mucin*-*type *O-*glycans were implicated in proteolytic activation and deactivation of glycoproteins such as peptide hormones, LDL-receptor, A disintegrin, and metalloprotease 17 (ADAM17), G protein coupled receptor and copper transporter [reviewed in (Schjoldager and Clausen, 2012)]. For instance, two *O-*glycans on peptidylglycine α-amidating monooxygenase (PAM) from rat controlled cleavage of a nearby furin protease recognition site. *O-*glycosylated PAM was localized in multivesicular bodies while the nonglycosylated form was rapidly degraded, implicating *O-*glycans in the recruitment of cargo into the endosomal pathway (Vishwanatha et al., 2016). Moreover, *O-*glycosylation controlled proteolytic events such as the down*-*regulation of the human fibroblast growth factor 23 (Tagliabracci et al., 2014) or the shedding of interleukin*-*2 receptor (Karabasheva et al., 2014). It should however be noted that a role of a glycan in protein fate control does not exclude additional roles of the same glycan, e.g. signalling. This is exemplified by the human peptide hormone atrial natriuretic peptide that was both stabilized *in vivo* by *O-*glycosylation as well as made to bind with higher affinity to its cognate receptor called natriuretic peptide receptor B (Hansen et al., 2019). In *Saccharomyces cerevisae*, *O-*linked mannosides protected several GPI-anchored proteins against the action of GPI-anchored aspartate proteases Yps1p/2p and it was suggested *O-*mannosylation might represent a dominant factor determining the stability of membrane proteins against proteolytic shedding activities (Dube et al., 2015). The large variety of yeast *O-*mannosylated proteins including 78% of all GPI-anchored proteins suggests an important regulatory role of *O-*glycosylation for many pathways (Neubert et al., 2016). This observation is intriguing because also in plants, many GPI-anchored proteins are predicted to carry type II AG (Borner et al., 2002). When the effect of *O-*mannosylation on protein fate was systematically tested for 137 (non GPI-anchored) *S. cerevisiae* proteins, 39 were found to be either stabilized or destabilized in mannosylation defective mutants (Castells-Ballester et al., 2018). This indicated that defective protein *O-*mannosylation does not indiscriminately lead to protein degradation but that this modification plays an active part in regulating protein fate during environmental stress and development. However, the potential analogy between *O-*mannosylation in yeast and AG-glycosylation in plants is limited by the fact that the former is initiated in the ER while the latter is a Golgi specific process. Hence, the mechanics of *O-*glycosylation dependent protein lifetime control might be different in these two kingdoms.

In addition to, and sometimes as a consequence of, altering protein maturation and turnover, *O-*glycosylation also affects the intracellular trafficking and localization of proteins. The resistance of secretion factor Tango1 from *Drosophila melanogaster* towards degradation by furin protease Dfur2 depended on its *O-*glycosylation, which in turn regulated the secretion of the adhesion protein Tiggrin (Zhang et al., 2010; Zhang and Ten Hagen, 2011; Zhang et al., 2014). In mice lacking an enzyme that initiates *O-*glycosylation, extracellular matrix components such as collagen and laminin failed to be secreted and instead, accumulated in the ER (Tian et al., 2012). In another study, the degree of GalNac *O-*glycosylation of the trans-membrane protein MUC1 determined the rate of its endocytosis in chinese hamster ovary cells, with higher rates of endocytosis and degradation experienced by the under-glycosylated form (Altschuler et al., 2000) and *O-*glycan remodelling of MUC1 in the recycling endosome of human epithelial kidney cells and breast cancer cells was suggested to control its subsequent fate (Razawi et al., 2013). Because of the many different GalNac-transferase encoding genes, it has been suggested that tissue- and substrate-specific *O-*glycosylation might be an important aspect of controlling the composition of the proteome of animal cells (Schjoldager and Clausen, 2012). It is intriguing to speculate that the multitude of GT31 genes in the *A. thaliana* genome, several of which have already been linked with type II AG biosynthesis, suggests functional analogy between the phylogenetically diverged *O-*glycosylation events in different kingdoms. The observation that synthetic AGPs are differentially *O-*glycosylated in various tissues supports this suggestion (Estevez et al., 2006). Compared to the relatively small branched mucin type glycans, linear glycosyaminoglycans (GAGs), that decorate mammalian proteoglycans, can well exceed hundreds of sugar subunits. The striking effects of GAG modification on protein export rate, polar sorting, regulated secretion, and sorting at the plasma membrane have recently been reviewed (Mihov and Spiess, 2015). To give an example, the amyloid precursor protein exists in both GAG-modified and -unmodified forms due to alternative splicing. Compared to the non*-*glycosylated form, that predominantly was targeted to the lysosome, the GAG-modified form was rapidly transported to the surface of human HeLa cells without lag (Mihov et al., 2015), an effect strikingly reminiscent of the increased secretion rate of *O-*glycosylated AGP21 and synthetic *O-*glycosylated recombinant proteins compared to non*-*glycosylated variant in plants (see above). The mechanism of how GAG modification affects trafficking remains to be elucidated but either the participation of cargo receptors or biophysical effects have been speculated to be involved (Mihov and Spiess, 2015).

## Conclusion and Perspectives

To summarize, in animals, in yeast and in plants, *O-*glycans are likely to be implicated in the regulation of proteolytic processing and sub-cellular targeting of numerous important glycoproteins, however, irrespective of the model system, a role for a particular glycan in protein fate control does not preclude additional functional roles of the same glycan such as supermolecular crosslinkers, cation reservoirs or as receptor ligands. In fact, multiple roles might coexist in order to prevent spurious effects of a signaling molecule (**Figure 2C**). While the field of *N-*glycosylation has strongly profited from inter-kingdom conservation, *O-*glycosylation research in plants can only draw analogies between different kingdoms and therefore we are still at a very early stage in our understanding of how this type of post-translational modification might act in the control of protein fate. One of the crucial questions with respect to elucidating the role of type II AG for protein fate is the identification of the sorting mechanisms that distinguish between different glyco- or aglycoforms of hybrid AGPs. The first hard challenge is the identification of cargo receptors involved in the process by genetic or biochemical approaches. Because type II AG is generated in the Golgi, the most likely localization of such receptors should be the trans-Golgi network also termed sorting endosome. Some cargo receptors are known to act in a lectin*-*like fashion such as the textbook example of the mannose-6-phosphate receptors (MPR) that recognize mannose-6-phosphate modified *N-*glycans on hydrolases at the trans-Golgi network to facilitate their transport into the lysosome (Castonguay et al., 2011). Another example of a lectin like secretory cargo receptor is mammalian ERGIC-53 that acts in the transport of *N-*glycosylated proteins from the ER to the Golgi (Hauri et al., 2000). However, so far, no *O-*glycan specific sorting receptors nor the identity of the involved cytosolic adaptor complexes are known in plants. Another possibility is that a low degree or absence of *O-*glycosylation might increasingly expose the intrinsic disorder in proline*-*rich regions (Shafee et al., 2020). Because intrinsically disordered protein regions are known to undergo a variety of molecular interactions in a fuzzy or plastic way (van der Lee et al., 2014), these regions might be recognized by novel cargo receptors. In conclusion to elucidate the potential role of type II AG modification for protein fate is an exciting new challenge for plant cell biology.

## Author Contributions

The author confirms being the sole contributor of this work and has approved it for publication.

## Funding

This work was supported by the Austrian Science Fund (FWF) grant number B32332-B.

## Conflict of Interest

The author declares that the research was conducted in the absence of any commercial or financial relationships that could be construed as a potential conflict of interest.

## Footnotes

1 Using the Patmatch tool of TAIR and a database of GO terms (TAIR10, GO terms extracellular, plasma membrane, cell wall), the motif [AVSTG]Pro[AVSTGA][GAVPSTC][APSDE] (where Pro is the modification site), is found in 1191 predicted *A. thaliana* loci (GJ Seifert, unpublished data-mining).
